# High-Frequency Generation of Homozygous/Biallelic Mutants via CRISPR/Cas9 Driven by *AtKu70*/*80* Promoters

**DOI:** 10.3390/ijms26189094

**Published:** 2025-09-18

**Authors:** Huihui Zhang, Chong Teng, Shanhua Lyu, Yinglun Fan

**Affiliations:** College of Agriculture and Life Science, Liaocheng University, Liaocheng 252000, China; 2310190206@stu.lcu.edu.cn (H.Z.); 2110190206@stu.lcu.edu.cn (C.T.)

**Keywords:** CRISPR/Cas9, homozygous/biallelic mutant, *AtKu70*/*80* promoter, hairy root transformation, *Rhizobium rhizogenes*-mediated transformation, *Rhizobium tumefaciens*-mediated transformation

## Abstract

CRISPR/Cas9 gene editing technology is widely used in plant gene editing to verify gene function or improve agronomic traits. In the CRISPR/Cas9 system, *Cas9* expression hinges on promoter choice, and CRISPR/Cas9 driven by a strong promoter or cell division-specific promoter has a higher editing efficiency. The CRISPR/Cas9 mechanism involves the CAS9 enzyme, which, directed by guide RNA, cleaves target double-stranded DNA and subsequently induces insertions or deletions (InDels) through the non-homologous end joining (NHEJ) repair pathway. The Ku protein plays a central role in the NHEJ repair process. It remains unclear whether driving *Cas9* with promoters of *AtKu70* and *AtKu80*, which are subunits of the Ku protein, will enhance gene editing efficiency. In this study, the promoters of *AtKu70* and *AtKu80* were cloned and used to drive *Cas9* in the CRISPR/Cas9 system. Four different genes, *GmRj7*, *GmNNL1*, *AtPDS3*, and *AtBRI1*, were designed for soybean hairy root transformation and *Arabidopsis* transformation. The results showed that the CRISPR/Cas9 systems driven by the promoters of *AtKu70* and *AtKu80* achieved higher homozygous/biallelic mutation efficiencies than the CRISPR/Cas9 system driven by the 35S promoter in hairy root transformation by *Rhizobium rhizogenes* and stable genetic transformation with *Rhizobium tumefaciens*.

## 1. Introduction

CRISPR/Cas9 gene editing technology has become the cornerstone of functional genomics and crop improvement, enabling precise gene validation, trait enhancement, and the rapid creation of elite germplasm. It now drives advances in yield, quality, disease and herbicide resistance, and other key agronomic attributes. [1]. At the same time, it is also widely used in gene regulation, multiple gene editing, and de novo domestication of wild plants [2,3,4,5]. The promoters responsible for the expression of both *Cas9* and single guide RNA (sgRNA) play a crucial role for achieving efficient genome editing in plants using the CRISPR/Cas9 system [4]. The promoters widely used to drive the Cas9 gene are the cauliflower mosaic virus (CaMV) 35S promoter, which is used in dicotyledonous plants, and the maize ubiquitin promoter, which is used in monocotyledonous plants in CRISPR/Cas9 gene editing vectors [5]. However, the editing efficiency of these two promoters in dicotyledonous and monocotyledonous plants is not high. The efficiency of CRISPR/Cas9 driven by the 35S promoter in generating homozygous/biallelic mutants (HBM) at the *AtBRI1* target site in *Arabidopsis thaliana* T1 transgenic plants is very low. In contrast, the efficiency of CRISPR/Cas9 driven by the Yao promoter reached 74.1% in generating HBM at the same target site [6]. *Yao* is a gene preferentially expressed in tissues with vigorous cell division, especially in embryos, blastocysts, endosperm, and pollen [7]. The YAO promoter also exhibits high gene editing efficiency in *Citrus* [8]. In *Arabidopsis*, the frequency of gene mutations has been enhanced when using the egg cell-specific *EC1.2* promoter and the gametophyte-expressed *SPL* promoter to drive *Cas9* [9,10]. Additionally, the efficiency of CRISPR/cas9-mediated gene editing in *Arabidopsis* was analyzed by using four different promoters, *DD45*, *Lat52*, *Yao*, and *CDC45*. The results showed that the *DD45* promoter could improve the frequency of sgRNA targeted gene knock-in and sequence replacement via homologous recombination (HR) at several endogenous sites in *Arabidopsis* [11]. The promoter of a calreticulin-like protein gene, PCE8, generated highly efficient gene editing in T0 plants [12]. The *AhUBQ4* (*Ubiquitin* gene) promoter was cloned from the peanut plant and applied to a CRISPR/Cas9 system. The CAS9-GFP driven by the *AhUBQ4* promoter not only showed a stronger GFP fluorescence signal, but also was superior to the 35S-driven system in terms of editing efficiency and diversity of mutation types [13]. The maize *dmc1* gene was highly expressed in callus and tassel, and the CRISPR/Cas9 system driven by the *dmc1* promoter efficiently generates HBM in maize [4]. The *Arabidopsis* γ-glutamylcysteine synthase (AtGCS) showed high activity in the cotyledons and main root, and the CRISPR/Cas9 system driven with the *AtGCS* promoter showed higher homologous/biallelic mutation efficiency than the CaMV 35S promoter in hairy root transformation in soybean, *Lotus japonicus*, and tomato plants [14]. A higher proportion of HBM directly increases the probability of detecting the expected phenotype in primary transformants (T1 *Arabidopsis* or T0 tomato, soybean, and related species). This is especially critical in hairy root transformation, where heterozygous edits rarely yield visible phenotypes and only homozygous or biallelic mutations reliably manifest detectable changes. The applications of the reported promoters in the CRISPR/Cas9 editing vectors have been summarized in Supplementary Table S1.

In the CRISPR/Cas9 system, CAS9 endonuclease has been shown to induce site-specific double-strand breaks (DSBs). DSBs are then repaired either through the non-homologous end joining (NHEJ) process or via HR mechanisms [15,16]. When repair is carried out through HDR, no mutations are generated. In contrast, the NHEJ pathway typically results in mutations, specifically insertions or deletions (InDels). NHEJ is an alternative DNA repair mechanism that competes with homology-directed repair (HDR). If the NHEJ pathway can be enhanced and the HDR pathway can be weakened, the editing efficiency of CRISPR will be improved [17,18]. Ku is a heterodimeric protein composed of 70 and 80 kDa subunits and involved in numerous cellular processes. As a central initial DNA end-binding factor in the NHEJ pathway, the Ku protein promotes the recruitment of downstream factors [19,20]. At the DSBs, Ku acts as a scaffold for the entire DNA repair complex, interacting both directly and indirectly with multiple NHEJ factors and processing enzymes. Through a central loop formed by the intertwined strands of its Ku70 and Ku80 subunits, Ku binds double-stranded DNA ends with high affinity in a sequence-independent manner—a structure that helps keep DNA ends in proximity [21]. Therefore, the Ku protein is essential for maintaining genome integrity and proper cell and tissue development, and its role is to bind and stabilize the ends of broken DNA molecules [19]. The expression analyses of *AtKu70* and *AtKu80* showed that they were highly expressed in the shoot apex and roots based on *Arabidopsis* Information Resource (TAIR) data (Supplementary Figure S1) [22]. Based on these studies, we speculate that the promoters of *AtKu70* and *AtKu80* have the potential to improve editing efficiency as drivers of the *Cas9* gene.

Here, we constructed the CRISPR/Cas9 vectors of the *Cas9* gene driven by the *AtKu70* and *AtKu80* promoters and introduced a red fluorescent selectable marker into the cloning site of gRNA to facilitate the editing efficiency. The editing efficiencies of the 35S, *AtKu70*, and *AtKu80* promoters were compared by *Rhizobium rhizogenes* (formerly known as *Agrobacterium rhizogenes*)-mediated hairy root transformation by targeting the *GmRj7* and *GmNNL1* genes in soybean. The *AtPDS3* and *AtBRI1* genes were selected in *Arabidopsis* for editing efficiency analysis with the three promoters by *Rhizobium tumefaciens* (formerly known as *Agrobacterium tumefaciens*)-mediated genetic transformation.

## 2. Results

### 2.1. Construction of pRd35Cas9-2BR, pRdKu70Cas9-2BR and pRdKu80Cas9-2BR

**Figure 1:**
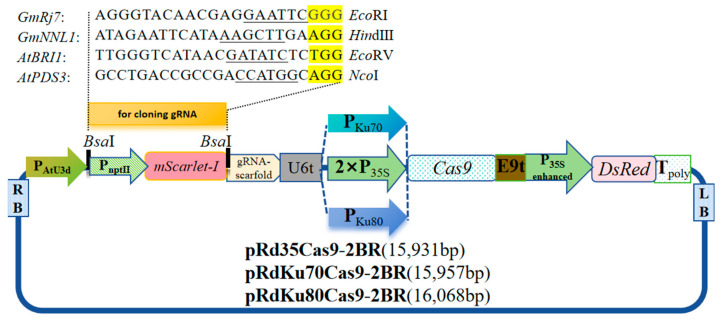
The backbone of CRISPR/Cas9 vectors. The P_NptⅡ_:*mScarlet-I* expression cassette was used for a visual selection marker in *E. coli*. The two *Bsa*I sites are designed for cloning gRNAs. The gRNAs were driven by the *AtU3d* promoter. The *AtKu70*, *AtKu80*, and 2×35S promoters drive the *Cas9* gene, respectively. The reporter gene, *DsRed*, is driven by the enhanced 35S promoter. The underlined sequences of target sites are recognition sequences of restriction endonucleases. The sequences highlighted with yellow are the PAM (Protospacer Adjacent Motif) sequences.

The *mScarlet-I* expression cassette was introduced between two *Bsa*I sites of the gene editing vector pRd35Cas9 [14] and replaced the *E. coli* selectable marker gene, resistance to spectinomycin and streptomycin. The new editing vector was named pRd35Cas9-2BR (Figure 1).

The promoters of *AtKu70* and *AtKu80* were amplified from *Arabidopsis* Col-0 and cloned in between the *Kpn*I and *Xba*I enzyme sites of pRd35Cas9-2BR, replacing the 2×35S promoter of pRd35Cas9-2BR. The two gene editing vectors were named pRdKu70Cas9-2BR and pRdKu80Cas9-2BR, respectively (Figure 1). The three gene editing vectors harboring the *mScarlet-I* expression cassette exhibited a red color in *E. coli* and could emit red fluorescence under green excitation light. When gRNAs were introduced between the two *Bsa*I sites, the *mScarlet-I* expression cassette was replaced, and positive recombinant colonies appeared white in *E. coli*.

### 2.2. Hairy Root Transformation and Gene Editing Efficiency of GmRj7, and GmNNL1

**Figure 2:**
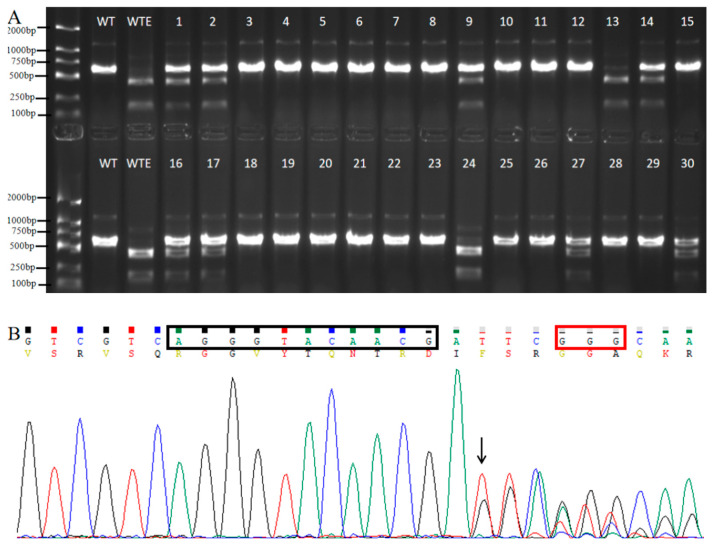
Verification of *GmRj7* target gene editing (**A**) *Eco*RI digestion electrophoresis of PCR products with the editing vector pRd35Cas9-Rj7. Lane WT: undigested PCR fragment; lane WTE: digested PCR fragment by *Eco*RI. Lanes 1–30: different independent transgenic hairy roots. (**B**) Sanger sequencing of PCR product undigested with *Eco*RI. The sequence in the black box is part of target site and the sequence in the red box is the PAM. The black arrow: starting from this base, the sequencing chromatogram shows mixed peaks.

*DsRed* serves as a plant reporter gene in these editing vectors; therefore, the hairy roots exhibiting red fluorescence are considered transgenic positive roots [14]. Genomic DNA was extracted from these transgenic positive roots, and the resulting PCR products were digested with restriction enzymes. If the PCR product was completely digested, the gene target of the transgenic root was unedited. If the PCR product was partially digested, the gene target of the transgenic root was a heterozygous edited type. If the PCR product remained undigested, the transgenic root was classified as a homozygous/biallelic mutant type. Analysis of the editing types of the *GmRj7* target site were performed using PCR products digested with *Eco*RI from 30 independent transgenic hairy root lines. The editing types of the *GmRj7* gene target site in hairy root transformation events mediated by the editing vector pRd35Cas9-Rj7 are shown in Figure 2A. The PCR products that could not be digested by *Eco*RI were verified by Sanger sequencing, and the results showed that base InDels (Insertions or Deletions) occurred near the PAM (Figure 2B). The editing types associated with the editing vectors pRdKu70Cas9-Rj7 and pRdKu80Cas9-Rj7 are shown in Supplementary Figure S2A,B.

To analyze the editing types of *GmNNL1*, a total of 90 independent transgenic hairy roots were used to digest the PCR product with *Hin*dIII (Supplementary Figure S3A–C). According to the results of *Hin*dIII endonuclease digestion, the editing efficiencies of CRISPR/Cas9 driven by these three promoters were significantly different (Table 1). The HBM editing efficiency of Cas9 driven by the *AtKu70* and *AtKu80* promoters reached 50%, while that of Cas9 driven by the 35s promoter was only 25%. The sequencing chromatogram showed that the editing events happened in the *GmNNL1* target site (Supplementary Figure S3D,E).

### 2.3. Validation of the Gene Editing Efficiency in Arabidopsis

**Figure 3:**
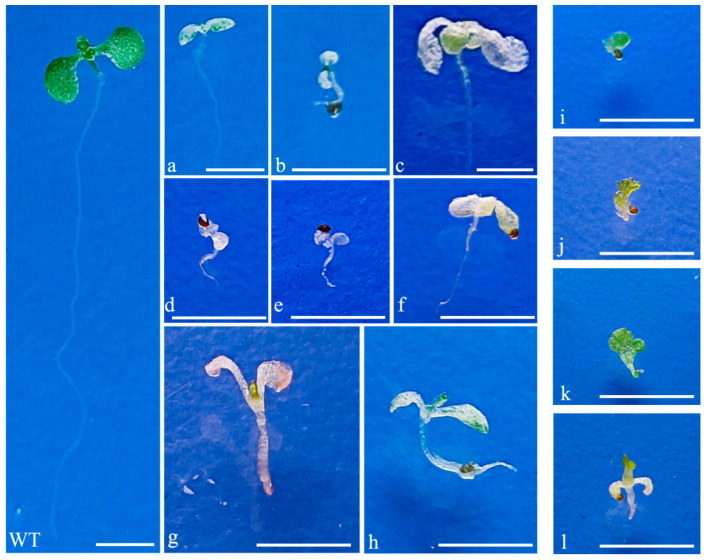
The albino or dwarf phenotypes after editing *AtPDS3* and *AtBRI1 in* Arabidopsis, respectively. (**a**–**h**): albino phenotypes; (**i**–**l**): dwarf phenotypes. Scale bars = 1 cm.

The *AtPDS3* gene encodes a phytoene desaturase enzyme in *Arabidopsis*, and the *pds3* mutant exhibits albino phenotypes [23]. The T1 seeds that could emit red fluorescence under green excitation light were transgenic-positive seeds. T1 seeds were used for germination on 1/2 MS medium. The numbers of T1 seedlings with albino phenotypes are shown in Table 2. The albino phenotypes at the seedling stage are shown in Figure 3a–h.

**Figure 4:**
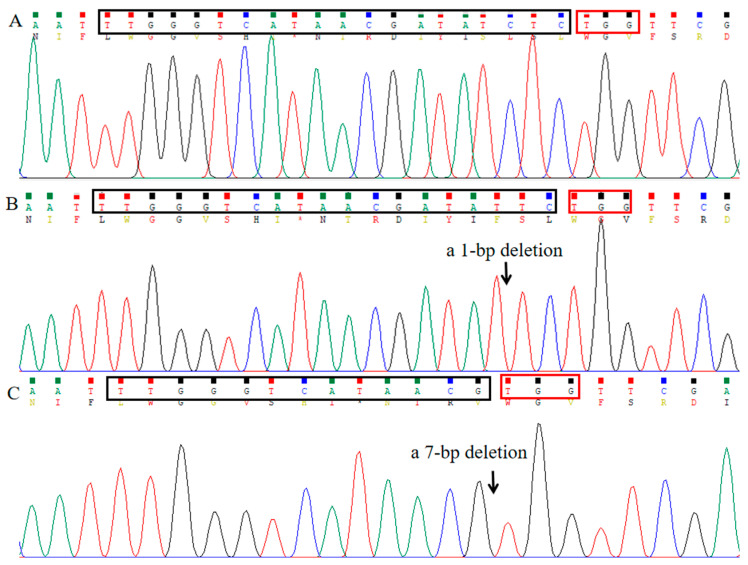
An example showing that knockout of *AtBRI* led to dwarf phenotypes at the seedling stage. (**A**): The Sanger sequencing map of *AtBRI* wild type. Sequence analysis revealed that this dwarf plant contained two mutant alleles, one with a 1 bp deletion (**B**) and another with a 7 bp deletion (**C**). The sequences in black boxes are the target site or part of the target site and the sequences in red boxes are the PAM sequences.

As *AtBRI1* loss-of-function plants exhibit dwarfism [24], plants with a dwarf phenotype in transgenic positive T1 plants are HBM (Figure 3i–k). The editing efficiency of CRISPR/Cas9 driven by the *AtKu70* and *AtKu80* promoters was 2.47-fold and 2.03-fold higher, respectively, than the system driven by the 35S promoter at the *AtPDS* gene locus. Similarly, at the *AtBRI1* gene locus, the editing efficiencies of CRISPR/Cas9 driven by *AtKu70* and *AtKu80* promoters were 2.45-fold and 1.95-fold higher, respectively, higher than the system driven by the 35S promoter (Table 2). Sanger sequencing of one plant with a dwarf phenotype showed the designed target site of *AtBRI1* contained two mutant alleles, one with a 1 bp deletion and another with a 7 bp deletion (Figure 4).

To determine whether *AtPDS* or *AtBRI1* had been edited in phenotypically normal plants, genomic DNA was extracted from normal T1 seedlings. After PCR amplification, the PCR products were digested with *Nco*I and *Eco*RV and resolved by electrophoresis (Supplementary Figures S4 and S5). The results revealed that a subset of these outwardly normal plants had edited target sites of *AtPDS* or *AtBRI1*, indicating heterozygous editing events that did not produce visible phenotypes. Analysis of the heterozygous editing efficiencies at both *AtPDS* and *AtBRI1* showed that *Cas9* driven by the *AtKu70* or *AtKu80* promoters outperformed the 35S promoter (Supplementary Table S2). Thus, when both homozygous and heterozygous edits are taken into account, the *AtKu70*/*AtKu80* promoters confer higher overall editing efficiencies than the 35S promoter.

## 3. Discussion

### 3.1. Simply and Effectively Selectable Marker in E. coli

Widely used editing vectors typically employ antibiotics resistance genes [25] or lethal genes (such as *ccdB*, control of cell division B) [5] as selectable markers, which requires adding antibiotics to the culture medium or using a special *E. coli* strain. The *mScarlet-I* expression cassette was employed as a negative selectable marker for selecting positive recombinants in *E. coli* in our previously constructed pBTR vectors. The positive recombinants can be visually distinguished from non-positive ones by the naked eye [26]. The *mScarlet-I* expression cassette was introduced between two *Bsa*I sites of the gene editing vectors pRd35Cas9-2BR, pRdKu70Cas9-2BR and pRdKu80Cas9-2BR in this study. It is simple, convenient, and effective to select positive recombinants using the negative selectable marker in gene editing vectors.

### 3.2. The CRISPR/Cas9 System Driven by AtKu70/80 Promoters Significantly Improves Proportion of Homozygous/Biallelic Mutation in R. rhizogenes and R. tumefaciens-Mediated Transformation

In our previous study, 30 independent transgenic roots were used to evaluate gene editing efficiency [14]. Accordingly, 30 transgenic roots were used to evaluate gene editing efficiency at the *GmRj7* target site in the present study. There was no significant difference in editing efficiency at the *GmRj7* target site with three different promoters. To more accurately assess the editing efficiency at the *GmNNL1* target site, 90 transgenic roots were utilized for this evaluation. The homozygous/biallelic editing efficiencies of CRISPR/Cas9 driven by the *AtKu70* and *AtKu80* promoters were twice that of the CaMV 35S promoter at the *GmNNL1* target site. The homozygous/biallelic editing efficiencies at the *AtPDS* and *AtBRI* target sites with CRISPR/Cas9 driven by the *AtKu70* promoter were 2.47 and 2.45 times, respectively, higher than that of the 35S promoter in *Arabidopsis.* Therefore, the gene editing systems driven by the *AtKu70* and *AtKu80* promoters were more efficient than the editing system driven by the 35S promoter. TAIR data show that AtKu70 and AtKu80 are broadly expressed in *Arabidopsis*, with particularly high activity in embryos, the apical meristem, flowers, and roots [27]. We speculate that, when CAS9 cleaves double-stranded DNA, the DSBs will further activate strong expression of the *AtKu70* and *AtKu80* promoters, so that the CRISPR/Cas9 editing system driven by the *AtKu70* or *AtKu80* promoters has high editing efficiency. In our previous study, the HBM generation efficiency at the same target of *GmRj7* using the CRISPR/Cas9 system driven by the *AtGCS* promoter was 70% [14], which indicated that there is little difference in HBM editing efficiency caused by different promoters at this same target of the *GmRj7* gene. However, at the same target of the *GmNNL1* gene, the editing efficiency of HBM with the *AtGCS* promoter was only 6.7% [14], while the efficiency with the Ku70 promoter reached 50%; the efficiencies of HBM generation with CRISPR/Cas9 driven by the promoters of *AtKu70/AtKu80* are higher than that of the *AtGCS* promoter at this same target of *GmNNL*. The HBM editing efficiency caused by the *Yao* promoter at the same target of *AtBRI1* was 71.4% [6], which is higher than those of *AtKu70/AtKu80*. The CRISPR/Cas9 driven by the *Yao* promoter generated 65%, 45.5%, and 75% HBM efficiencies, respectively, with three different targets of the PDS gene in *citrus* [8]. The efficiency of the maize *dmc1* promoter in generating HBM in maize was 66% [4]. The reports mentioned above show that the efficiencies of generating HBM in the CRISPR/Cas9 systems driven by these promoters were higher than that driven by the 35S promoter. The method of calculating the gene editing efficiency of HBM based on changes in phenotypes is the simplest, but it is only limited to phenotypes that can be directly observed with the naked eye and cannot detect heterozygous editing events. In contrast, methods based on deep sequencing [28] and next-generation sequencing (NGS) [29] can detect all types of sequence changes in the edited region, such as insertions/deletions (indels), base substitutions, and inversions.

CRISPR/Cas9 gene editing has become a prominent and widely used technique in plant research. Optimizing the CRISPR system to enhance editing efficiency remains a key focus in gene editing research. For example, the editing efficiency can be improved by modifying the promoter driving *Cas9* or gRNA, or by using codon-optimized *Cas9* [30]. Additionally, increasing the expression level and stability of the CAS9 protein, as well as improving the efficiency of CAS9 nuclear entry, also contributes to improving editing efficiency. When CAS9 is fused with six nuclear localization signal (NLS) peptides and two short peptides TAT and HA2 that help to penetrate the cell membrane, the gene editing efficiency is significantly enhanced [31]. Different NLS can also affect gene editing efficiency. A comparison of six types of NLS revealed that double-BP NLS exhibits the highest targeting efficiency [32]. Additionally, co-expression of a human RNA m6A demethylase (which promotes chromatin opening) in gene editing vectors significantly enhances the efficiency of gene editing in soybean and rice [33]. CRISPR/Cas9 technology enables simultaneous targeting of multiple genes, making it highly significant for studying the interaction of genes [34]. Notably, in polyploid plants, where some genes exhibit functional redundancy, simultaneous targeting of multiple genes is crucial for analyzing gene function. Moreover, a CRISPR/Cas9 system with high editing efficiency can be more effectively applied to multi-gene editing.

## 4. Materials and Methods

### 4.1. Plant Materials and Growth Conditions

Soybean variety Williams 82 and *Arabidopsis thaliana* Columbia (Col-0) were used in this study. The plants were grown in a greenhouse under a 16 h light/8 h dark photoperiod at 24 ± 2 °C. Restriction enzymes and T4 ligase were purchased from New England Biolabs Company (Ipswich, MA, USA). The oligo DNAs and primers were synthesized by Sangon Biotech Co., Ltd. (Shanghai, China). Taq PCR Master Mix (2×, with Blue Dye) carrying Taq DNA polymerase was purchased from Sangon Biotech Co., Ltd. (Shanghai, China).

### 4.2. Construction of Basic Editing Vectors

To facilitate the screening target gRNA insertion of positive recombinants in *E. coli*, the *mScarlet-I* expression cassette, comprising the promoter of the *neomycin phosphotransferase* gene (P_NptⅡ_), *mScarlet-I* coding domain sequence, and lambda T0 terminator, was introduced between two *Bsa*I sites of the editing vector pRd35Cas9 and allowed the construct to emit bright red fluorescence in *E. coli* [14]. PCR amplification was performed using primer set RdBsa1/RdBsa2 (all primer sequences used in this paper are listed in Supplementary Table S3) and with pMRE-Tn5-155 (Addgene Plasmid #118537) as the template [35]. The PCR product and pRd35Cas9 were mixed with *Bsa*I, T4 ligase in 1×T4 ligation buffer at 37 °C/30 min, 4 °C/60 min. The resulting mixture was transformed into chemically competent *E. coli* DH5α cells, and the red-color colonies were selected for restriction enzyme digestion verification.

To isolate the promoters of *AtKu70* (AT1G16970) and *AtKu80* (AT1G48050), 1540 bp and 1647 bp upstream were amplified from Col-0 genomic DNA via PCR using primer sets, At1g97K/At1g97X, At1g05K/At1g05X, which contain a *Kpn*I and *Xba*I sites, respectively. The PCR fragments and the newly constructed editing vector pRd35Cas9-2BR were digested with *Kpn*I and *Xba*I sites, respectively, and then ligated with T4 ligase in 1×T4 ligation buffer. The resulting editing vectors were named pRdKu70Cas9-2BR and pRdKu80Cas9-2BR, respectively.

### 4.3. Construction of Editing Vectors for Knockout GmRj7, GmNNL1, AtBRI1, and AtPDS3 Genes

The sequences and positional information of these four target sites of *GmRj7*, *GmNNL1*, *AtPDS3*, and *AtBRI1* and the recognition sites of restriction endonucleases are shown in Supplementary Figure S6. Four sets of oligo DNA, KtRj71/KtRj72, KtNNL1/KtNNL2, KtPD1/KtPD2, and KtBR1/KtBR2, were designed and used for construction of editing vectors for the knockout *GmRj7, GmNNL1, AtPDS3*, and *AtBRI1* genes, respectively. For inserting the sgRNA into *Bsa*I sites of editing vectors, the pair of two oligo DNAs were annealed at 60 °C for 5 min, and then cloned between two *Bsa*I sites of pRd35Cas9-2BR, pRdKu70Cas9-2BR, and pRdKu80Cas9-2BR according to one digestion–ligation reaction [14]. The positive clones did not show red fluorescence and white color [26]. A total of twelve editing vectors were constructed, and the gRNAs were sequenced with Sanger Sequencing.

### 4.4. Hairy Root Transformation in Soybean and Validation of Genome Editing Efficiency

The constructed editing vectors harboring the soybean editing sites were introduced into *R. rhizogenes* strain K599 by electroporation. Soybean variety Williams 82 was used for one-step hairy root transformation with *R. rhizogenes* K599 harboring the six editing vectors according to our previous report [36]. Thirty seedlings were used for hairy root transformation with pRd35Cas9-Rj7, pRdKu70Cas9-Rj7, and pRdKu80Cas9-Rj7, respectively. Ninety seedlings were used for hairy root transformation with pRd35Cas9-NNL1, pRdKu70Cas9-NNL1, and pRdKu80Cas9-NNL1, respectively.

The editing vectors used in this study harbor the *DsRed* reporter gene [14]. So the transgenic positive hairy roots were distinguished from nontransgenic hairy roots by visual DsRed fluorescence using a handheld LUYOR-3415RG fluorescent lamp. Each hairy root was regarded as an independent editing event; the primary hairy root that came from a callus of an explant was named the 1st generation hairy root and the branches of the 1st generation hairy root were named the 2nd generation hairy roots. In the study on gene editing in hairy roots of *Medicago truncatula*, it was found that 2nd generation hairy roots have a higher editing efficiency than 1st generation hairy roots [37]. Genomic DNA was extracted from transgenic positive 1st generation hairy roots when the length of transgenic hairy roots was up to 5 cm. There were very few 2nd generation hairy roots on the 1st generation hairy roots with a length of about 5 cm (Supplementary Figure S7). Thirty and ninety transgenic hairy roots were used for genomic DNA extraction with each editing vector of *GmRj7* and *GmNNL1*, respectively. Whether the target gene was edited was verified by enzyme digestion of the PCR product of the sequence where the target site was located. The primer sets Rj71/Rj72 and GmRHin1/GmRHin2 were used to amplify the target sequences of *GmRj7* and *GmNNL1*, respectively. The PCR products of the *GmRj7* target and *GmNNL1* target were subjected to *Eco*RI and *Hin*dIII, respectively.

### 4.5. Arabidopsis Transformation and Validation of Genome Editing Efficiency

The editing vectors pRd35Cas9-PDS, pRd35Cas9-BRI, pRdKu70Cas9-PDS, pRdKu70Cas9-BRI, pRdKu80Cas9-PDS, and pRdKu80Cas9-BRI were introduced into the *R. tumefaciens* strain GV3101 by electroporation. These editing vectors were transformed into wild-type *A. thaliana* Col-0 by the floral dip method.

Transgenic *Arabidopsis* was selected by visual DsRed fluorescence using a handheld LUYOR-3415RG fluorescent lamp because those editing vectors carried the *DsRed* reporter gene. When the edited gene was a heterozygous or homozygous double mutation, the phenotype of T1 *Arabidopsis* was directly observed. T1 seedlings exhibiting albino or dwarf phenotypes were counted to quantify HBM efficiency. To further confirm that T1 seedlings were not exhibiting albino or dwarf phenotypes, genomic DNAs were extracted from normal plants. The designed target sequence was amplified with the primer sets AtPDS3/AtPDS4 and AtBRIF/AtBRIR, and the PCR products were digested with *Nco*I and *Eco*RV to verify whether the target sites at *AtPDS3* and *AtBRI1* had been edited, respectively. The PCR products of individual plants with dwarf phenotype were cloned into the vector pYFRed [38], and 5 positive clones were randomly selected for Sanger sequencing to clarify the gene-editing types of the *AtBRI1* target.

## 5. Conclusions

This study focuses on the impact of promoters driving *Cas9* in the CRISPR/Cas9 system on the efficiency of plant gene editing. Given that the Ku protein plays a core role in NHEJ repair, it remains unclear whether the promoters of *AtKu70* and *AtKu80* can enhance editing efficiency when driving *Cas9*. The promoters of *AtKu70* and *AtKu80* were cloned to drive *Cas9* and performed gene editing on four targets, the soybean genes *GmRj7* and *GmNNL1* and the *Arabidopsis* genes *AtPDS3* and *AtBRI1*. The results showed that, in CRISPR/Cas9 systems driven by the *AtKu70* and *AtKu80* promoters, among the four designed targets, the editing efficiencies at the targets of three genes, namely *GmNNL1*, *AtPDS3*, and *AtBRI1*, were significantly higher than those of the system driven by the 35S promoter.

## Figures and Tables

**Table 1:** Editing efficiencies at target site in *GmRj7* and *GmNNL1* of different promoters.

CRISPR/Cas9 System	*GmRj7* (Number of HBM/Total)	*GmNNL1* (Number of HBM/Total)
pRd35Cas9-2BR	66.7% (20/30)	25.6% (23/90)
pRdKu70Cas9-2BR	66.7% (20/30)	50% (45/90)
pRdKu80Cas9-2BR	60% (18/30)	48.9% (44/90)

**Table 2:** Editing efficiencies at *AtPDS* and *AtBRI* in *Arabidopsis thaliana* of different promoters.

CRISPR/Cas9 System	*AtPDS* (Number of HBM/Total)	*AtBRI1* (Number of HBM/Total)
pRd35SCas9-2BR	19.2% (11/52)	17.7% (8/45)
pRdKu70Cas9-2BR	47.5% (29/61)	43.4% (23/53)
pRdKu80Cas9-2BR	39.3% (22/56)	34.5% (19/55)

## Data Availability

All data supporting the conclusions of this article are included in this article.

## Supplementary Materials

The following supporting information can be downloaded at https://www.mdpi.com/article/10.3390/ijms26189094/s1.
