# Supplementary material for: High-Frequency Generation of Homozygous/Biallelic Mutants via CRISPR/Cas9 Driven by AtKu70/80 Promoters
Source: Int J Mol Sci. 2025 Sep 18;26(18):9094. doi: 10.3390/ijms26189094 (PMC12469801; doi:10.3390/ijms26189094)
Supplement: Supplementary file 1 [file ijms-26-09094-s001.zip › ijms-3770480-supplementary.pdf]

Table S1. The different promoters drive *Cas9* in CRISPR/Cas9 system.

promoter	editing efficiency	Used in	references
35S promoter	35.6%	dicotyledonous plants	Ma et al., 2015
maize *ubiquitin* promoter	85.4%	monocotyledonous plants	Ma et al., 2015
Yao promoter	90.5%	*Arabidopsis*	Yan et al., 2015
	55%	*Citrus*	Zhang et al., 2017
EC1.2 promoter	8.3-24.8% HBM	*Arabidopsis*	Wang et al., 2015
SPL promoter	70% （T2）	*Arabidopsis*	Mao et al., 2016
Lat52 promoter	-	*Arabidopsis*	Mao et al., 2016
DD45 promoter	-	*Arabidopsis*	Mao et al., 2016<br>Miki et al., 2018
CDC45 promoter	-	*Arabidopsis*	Miki et al., 2018
AhUBQ4 promoter	--	hairy root transformation in peanut	Cui et al., 2025
PCE8 promoter	56-90%	tobacco	Li, et al. 2025
dmc1 promoter	66% HBM	*maize*	Feng et al., 2018
AtGCS promoter	76.7-81.3% HBM	hairy root transformation in soybean, *Lotus japonicus* and tomato	Liu et al., 2022

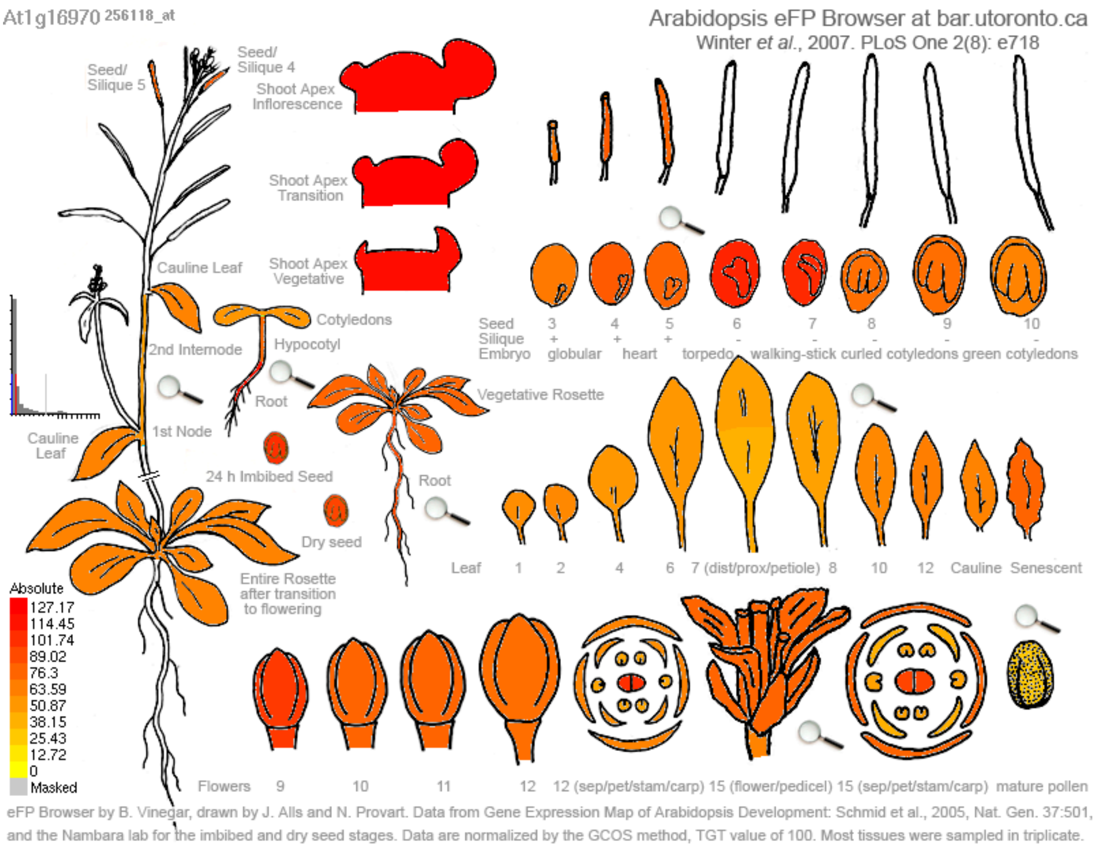

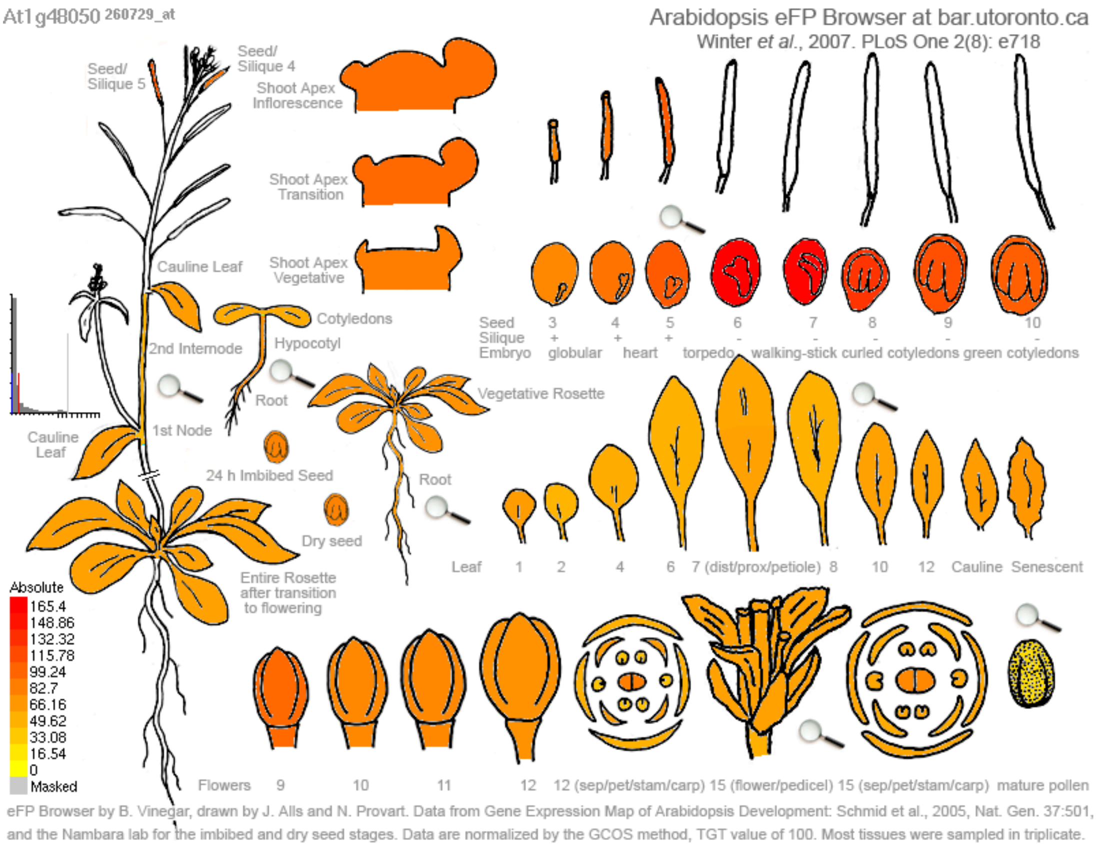

Figure S1. Expression analyses of *AtKu70* (AT1G16970) and *AtKu80* (AT1G48050). The data were come from TAIR (https://www.arabidopsis.org/).

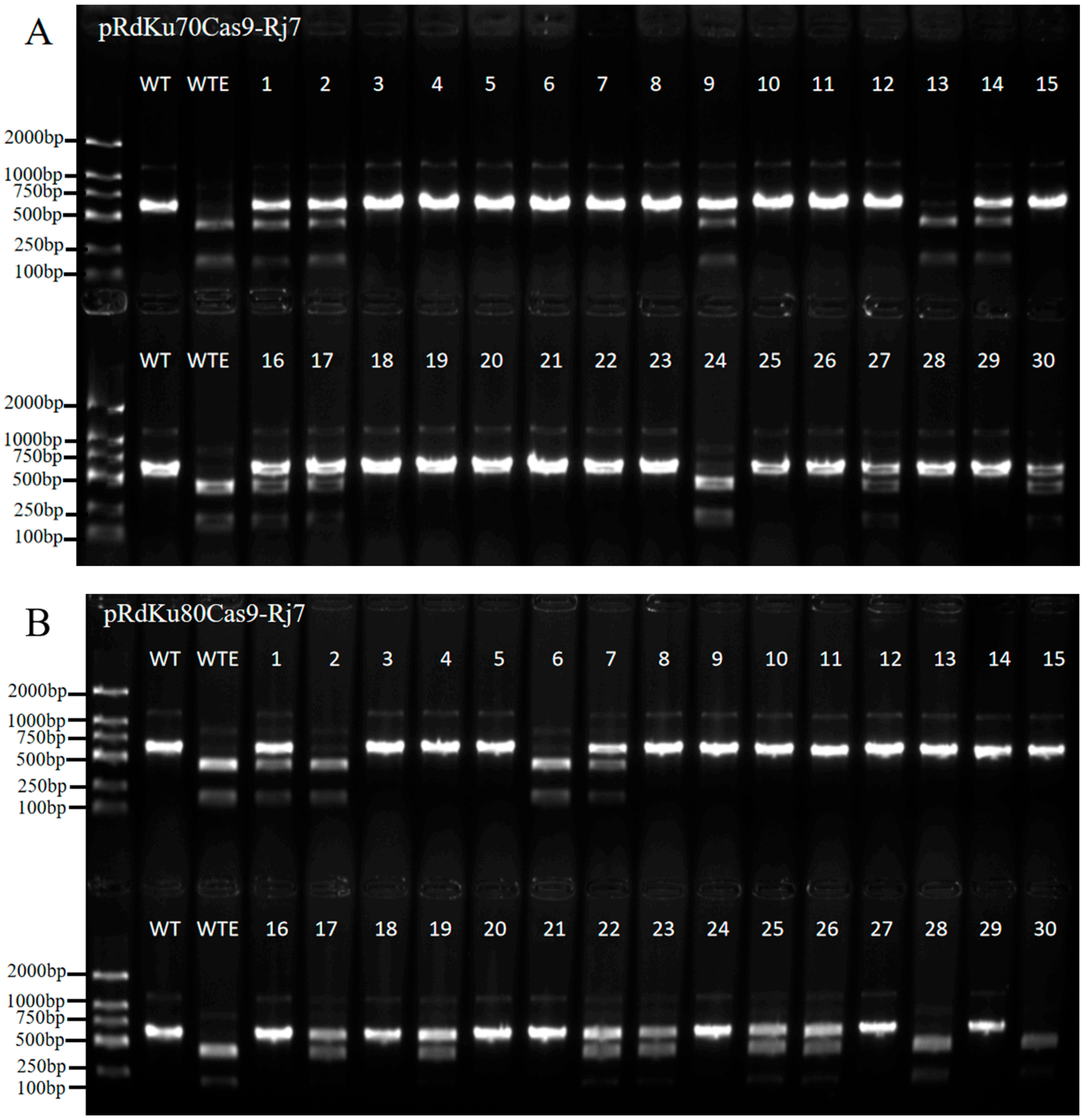

Figure S2. Enzyme digestion electrophoresis of PCR products with different targets.

(A) and (B) are corresponding to the editing pRdKu70Cas9-Rj7 and pRdKu80Cas9-Rj7, respectively. Lane WT: undigested PCR fragment; Lane WTE: digested PCR fragment by *Eco*RI. Lanes 1-30: different independent transgenic hairy roots.

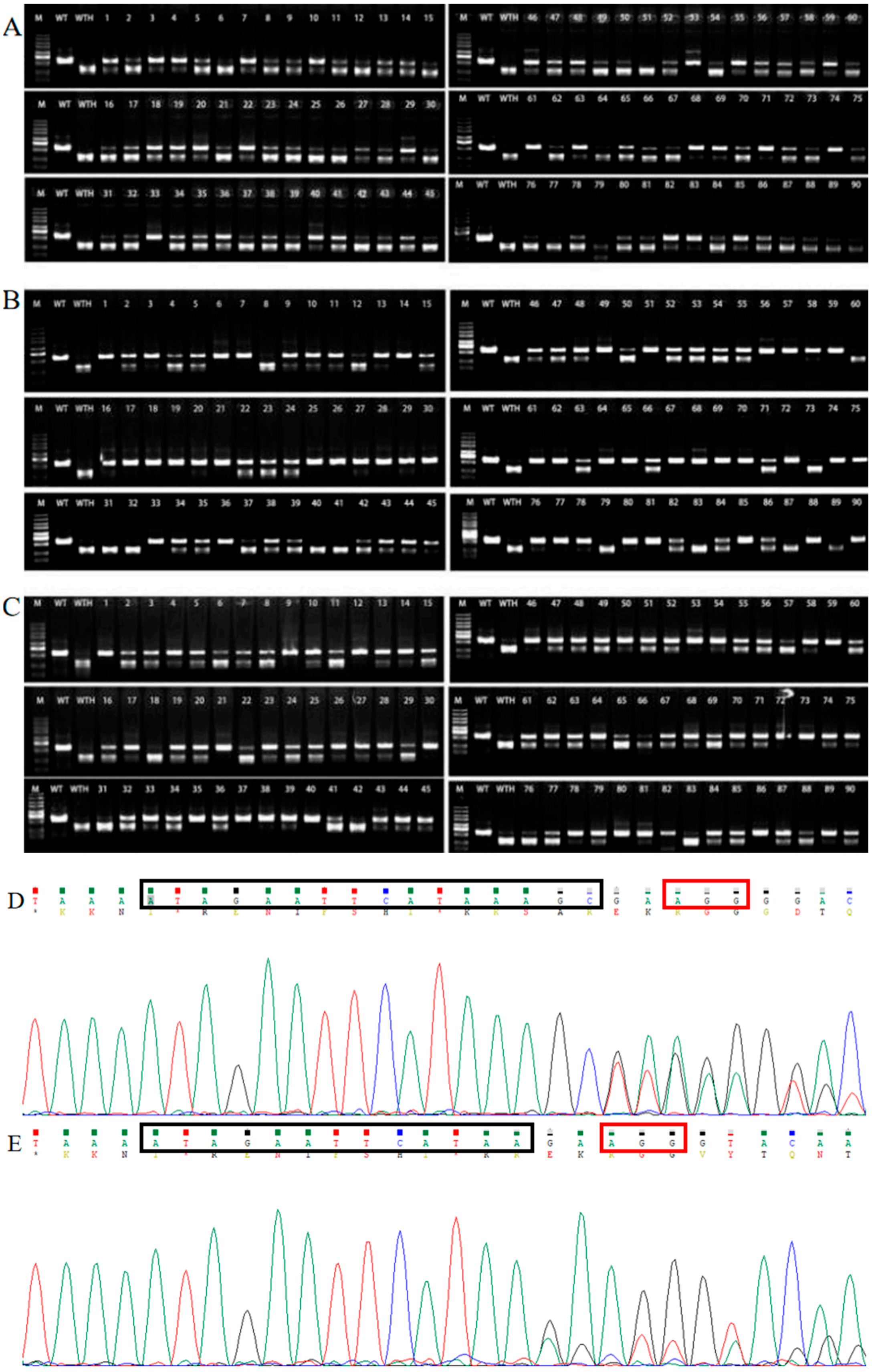

Figure S3. Enzyme digestion electrophoresis of PCR products with different targets. (A), (B), and (C) are corresponding to the editing pRd35Cas9-NNL1, pRdKu70Cas9-NNL1, and pRdKu80Cas9-NNL1, respectively. Lane WT: undigested PCR fragment; Lane WTH: digested PCR fragment by *Hin*dIII. Lanes 1-90: different independent transgenic hairy roots. (D-E), Sanger sequencing from two independent transgenic hairy roots.

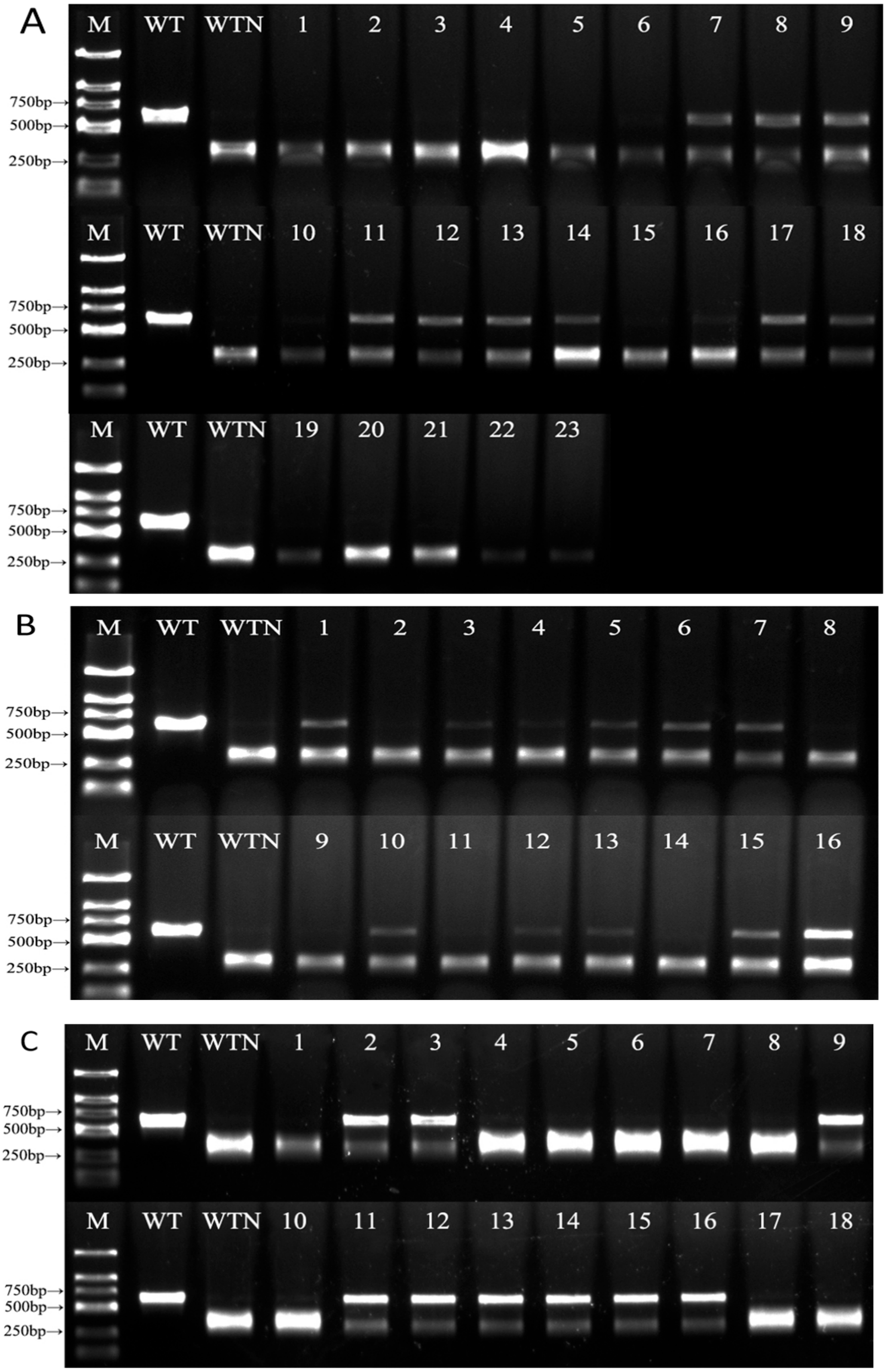

Figure S4. *Nco*I enzyme digestion electrophoresis of PCR products. (A), (B), and (C) are corresponding to the editing pRd35Cas9-PDS, pRdKu70Cas9-PDS and pRdKu80Cas9-PDS, respectively. Lane WT: undigested PCR product; Lane WTN: digested PCR fragment by *Nco*I. Lanes 1-23 (16 or 18): different independent transgenic lines

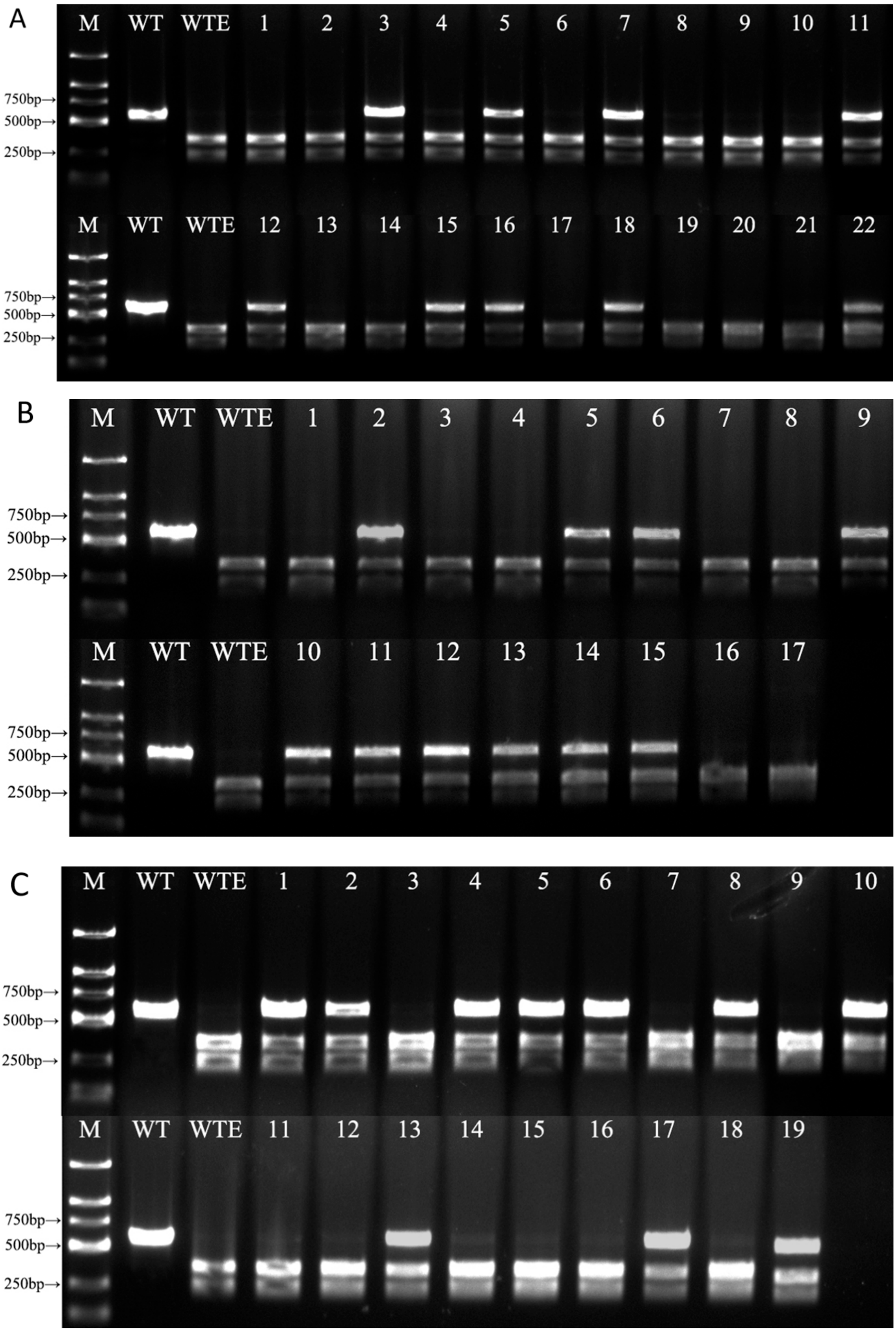

Figure S5. *Eco*RV enzyme digestion electrophoresis of PCR products. (A), (B), and (C) are corresponding to the editing pRd35Cas9-BRI, pRdKu70Cas9-BRI and pRdKu80Cas9-BRI, respectively. Lane WT: undigested PCR product; Lane WTE: digested PCR fragment by *Eco*RV. Lanes 1-22 (17 or 19): different independent transgenic lines

Table S2. Heterozygous editing efficiency of *AtPDS* and *AtBRI* in phenotypically normal *A. thaliana* with different promoters.

CRISPR/Cas9 system	*AtPDS* (Number of Heterozygous editing plants/ total)	*AtBRI1* (Number of Heterozygous editing plants/ total)
pRd35SCas9-2BR	39.1% (9/23)	40.9% (9/22)
pRdKu70Cas9-2BR	68.8% (11/16)	58.8% (10/17)
pRdKu80Cas9-2BR	50% (9/18)	52.6% (10/19)

Table S3. Oligo DNAs and Primers used in this study.

Name	Sequences (5'-3')<br>(The underlined sequences are the restriction sites used)
RdBsa1	CGTCATGTGTCAGGAGACCTTTTAGAGCTCGCACGCTG
RdBsa2	TAGCTCTAAAACCGAGACCTAGCTTGGATTCTCACCAAT
At1g97K	CGGGGTACCAAGTCAGGCTATGTATGGACGC
At1g97X	GGACTCTAGACAAGAACACGGATACAGTTATCG
At1g05K	CGGGGTACCGGGTTTGGTTTCTGTGTTGG
At1g05X	GTGTGTCTAGAGGAGTTGTGCTGGAAGAAG
KtRj71	GTCAAGGGTACAACGAGGAATTC
KtRj72	AAACGAATTCCTCGTTGTACCCT
KtNNL1	GTCATAGAATTCATAAAGCTTGA
KtNNL2	AAACTCAAGCTTTATGAATTCTA
KtPD1	GTCAGCCTGACCGCCGACCATGGC
KtPD2	AAACGCCATGGTCGGCGGTCAGGC
KtBR1	GTCATTGGGTCATAACGATATCTC
KtBR2	AAACGAGATATCGTTATGACCCAA
AtPDS3	GACGTCAGGAAGAACATGGTC
AtPDS4	GACATGGCAATAAACACCTCG
AtBRIF	GGCGAATTACCGGAATCTCT
AtBRIR	AGTAAGAGCTGACATAGCCTGAGG

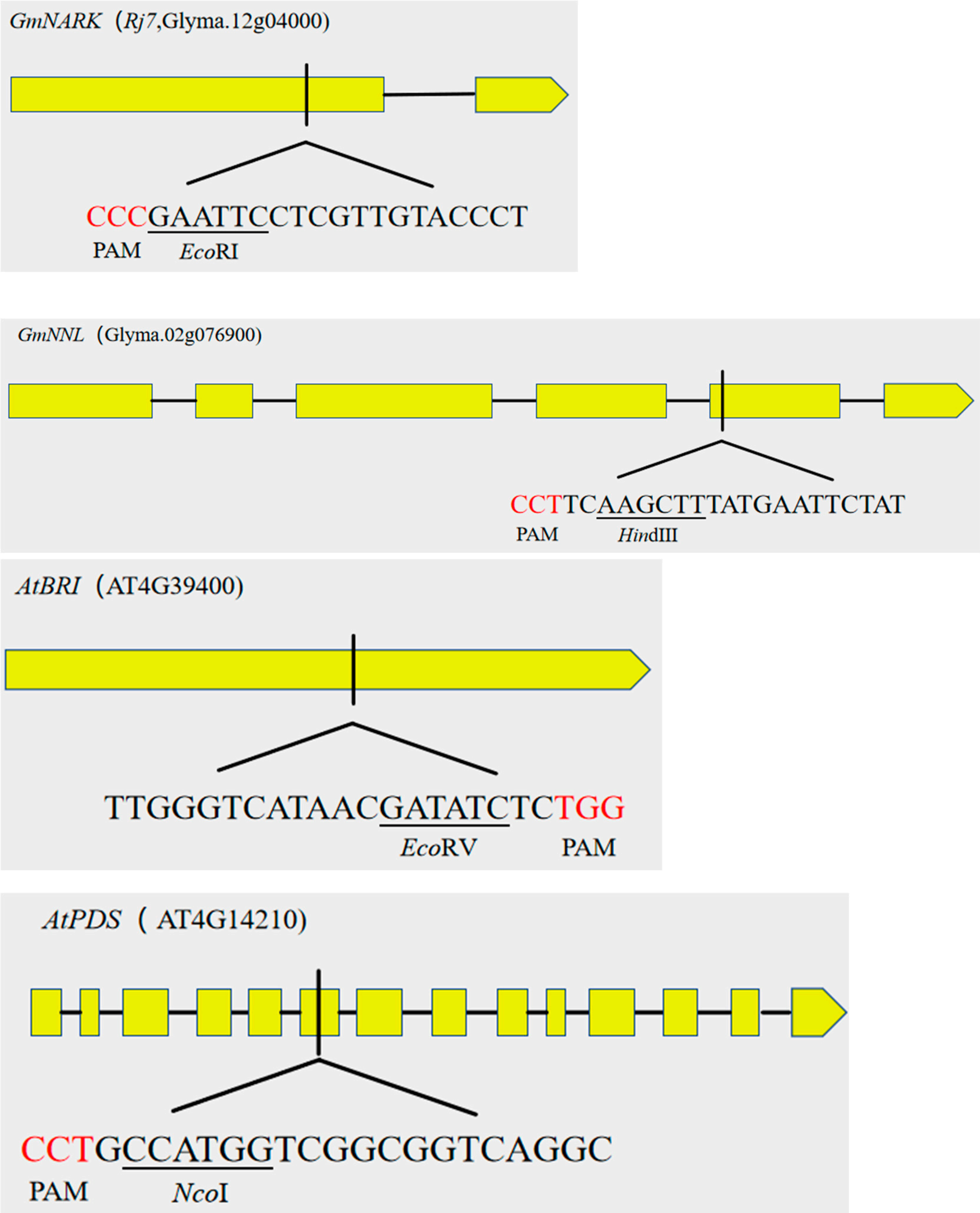

Figure S6. The positional information of the four target sites. The yellow boxes represent the exon of theses genes. The vertical line indicates the position of the target site. The red colored letters are the PAMs ot the target, and the underlined sequence is the restriction recognition site of the corresponding enzyme.

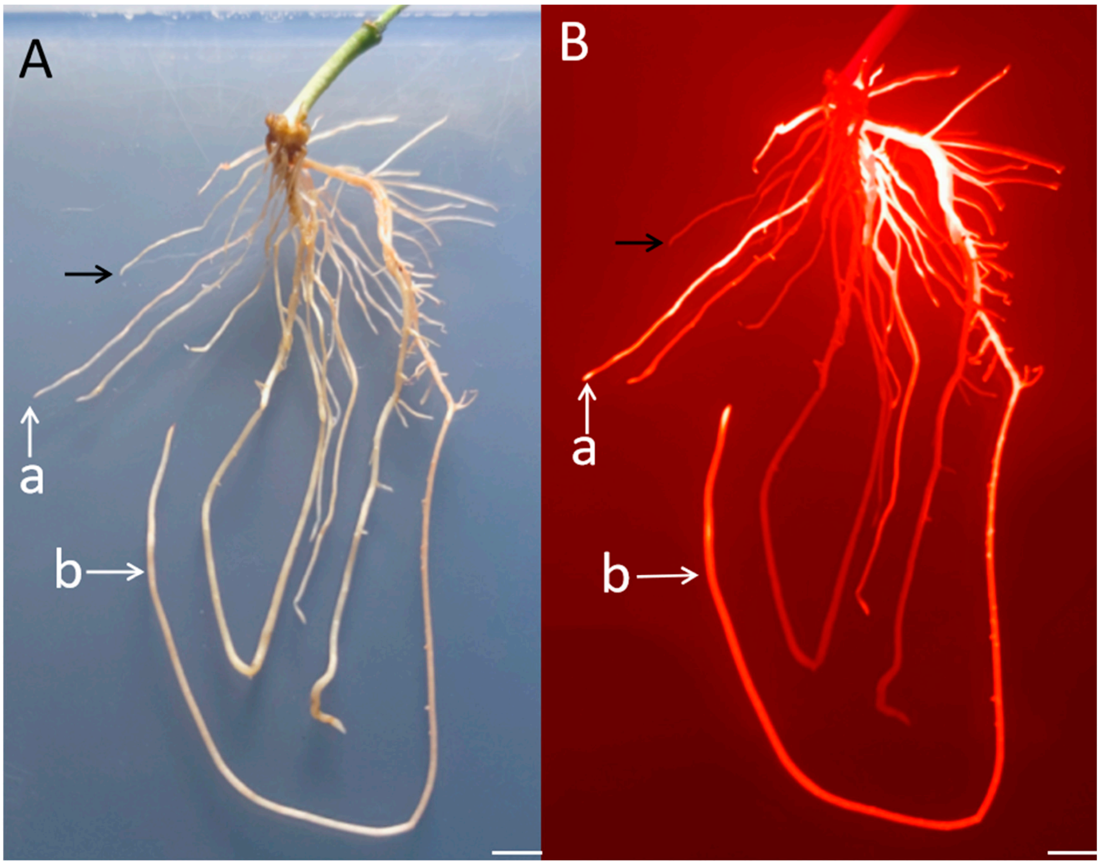

Figure S7. The hairy root formation in soybean via one-step hairy roo transformation with *R. rhizogenes* K599. A: under natural light; B: under green excitation light. Black arrows: non-transgenic root. White arrows: transgenic positive roots. a: roots with a length of about 5 cm are used for genomic DNA extraction. b, roots that are too long cannot be used for genomic DNA extraction. Scale bars = 1 cm.
